# Building a Multi-Institutional and Interdisciplinary Team to Develop a Zoonotic Tuberculosis Roadmap

**DOI:** 10.3389/fpubh.2018.00167

**Published:** 2018-06-12

**Authors:** Francisco Olea-Popelka, Paula I. Fujiwara

**Affiliations:** ^1^Mycobacteria Research Laboratories, Department of Clinical Sciences, College of Veterinary Medicine and Biomedical Sciences, Colorado State University, Fort Collins, CO, United States; ^2^International Union Against Tuberculosis and Lung Disease, Paris, France

**Keywords:** zoonotic, tuberculosis, roadmap, public, health, integrated, approach

## Abstract

Tuberculosis (TB), as the major infectious disease in the world, has devastating consequences for not only humans, but also cattle and several wildlife species. This disease presents additional challenges to human and veterinary health authorities given the zoonotic nature of the pathogens responsible for the disease across species. One of the main public health challenges regarding zoonotic TB (ZTB) caused by Mycobacterium bovis is that the true incidence of this type of TB in humans is not known and is likely to be underestimated. To effectively address challenges posed by ZTB, an integrated One Health approach is needed. In this manuscript, we describe the rationale, major steps, timeline, stakeholders, and important events that led to the assembling of a true integrated multi-institutional and interdisciplinary team that accomplished the ambitious goal of developing a ZTB roadmap, published in October, 2017. It outlines key activities to address the global challenges regarding the prevention, surveillance, diagnosis, and treatment of ZTB. We discuss and emphasize the importance of integrated approaches to be able to accomplish the short (year 2020) and medium term (year 2025) goals outlined in the ZTB roadmap.

## Introduction

Worldwide, there is consensus that solutions to complex issues need the participation and involvement of different stakeholders. Recent outbreaks of emerging and re-emerging zoonoses [i.e., Zika, Ebola, Middle East Respiratory Syndrome (MERS), Avian and Swine Influenza] (1) have heightened the public's awareness about the close and complex interrelationship between the health of humans, wildlife species, and domestic animals. Tuberculosis (TB), is one such disease that continues to have devastating consequences for not only humans, but also cattle and several wildlife species (2–5). Caused by bacteria belonging to the *Mycobacterium tuberculosis* complex (MTBC), TB presents additional challenges to human and veterinary health authorities given the zoonotic nature of the pathogens responsible for the disease and the ability of MTBC agents to be shared across species (4, 5). TB in humans is caused primarily by *Mycobacterium tuberculosis* (*M. tb*); worldwide, it is the leading cause of death in humans by an infectious disease (2). Bovine TB caused by *Mycobacterium bovis* (*M. bovis*) is widely distributed around the world (3, 6) and continues to cause considerable economic losses to farmers and countries due to the reduced production of affected animals, culling of animals from herds (or entire herd depopulation in some cases), and the elimination of affected (or all) parts of animal carcasses at slaughter (7). *M. bovis* can also infect and cause TB in humans (zoonotic tuberculosis (ZTB) (8–11). The World Health Organization (WHO) estimated that “in 2016 there were 147,000 new cases of ZTB and 12,500 deaths due to this type of TB” (2). Furthermore, *M. bovis* has the ability to cause TB and cause death in several wildlife species (4, 5).

One of the main public health challenges regarding ZTB is that its true incidence in humans is not known and is likely to be underestimated due to the lack of systematic surveillance for *M. bovis* as a causal agent of TB in people in all low-income, high TB burden countries where bovine TB is endemic, and the inability of laboratory procedures most commonly used to diagnose human TB to identify and differentiate *M. bovis* from *M. tb* (12). To effectively address challenges posed by ZTB (and other diseases at the “animal-human” interface), a cross-sectorial and multidisciplinary One Health approach linking animal, human, and environmental health is required.

In this manuscript, we describe the rationale, major steps, timeline, stakeholders, and important events that lead to the assembling of a true integrated multi-institutional and interdisciplinary team that worked toward and accomplished the ambitious goal of developing a ZTB roadmap that was published in English, Spanish, and French (13–15) to address the global challenges regarding the prevention, surveillance, diagnosis, and treatment of zoonotic TB (ZTB), globally. We discuss and emphasize the importance of integrated approaches to be able to accomplish the short (year 2020) and medium term (year 2025) goals outlined in the ZTB roadmap.

### A robust interdisciplinary platform

The International Union Against Tuberculosis and Lung Disease (The Union) is an international scientific organization that works with partners, including governments, academia, and civil society, to fight TB, tobacco use and other lung diseases in low- and middle-income countries, through technical assistance, training, and research. Through its volunteer members, it houses scientific sections that promote areas of specific interest. The Union's ZTB sub-section is a global network of physicians, veterinarians, researchers, economists and social anthropologists that works to understand the dynamics of ZTB, create global awareness, and facilitate multi-institutional collaboration to address the challenges posed by it. Efforts conducted by the ZTB sub-section has led to a continuous and stable increase in the number of activities, attention, and attendees to ZTB-related activities at the annual Union World Conference on Lung Health. For example, at the 2010 conference in Berlin, Germany, there was only one ZTB session (symposium) attended by less than 10 people. Over the last seven years, the ZTB activities at the annual conference have increased to two scientific symposia, one poster session, one meet the expert session, press releases, and a keynote talk on ZTB at the plenary session in South Africa during the 2015 conference. During the last conference in Guadalajara, Mexico in October 2017, an audience of approximately 75 professionals from different disciplines attended each of the two ZTB scientific symposia.

Prior to The Union's initial activities to create global awareness of ZTB, in 2010, the WHO, OIE and FAO pioneered One Health approaches under a tripartite partnership, which shares responsibilities and jointly develop and implement integrated strategies for addressing health risks at the human animal-ecosystem interface (12). The combined involvement and commitment of these three institutions has been crucial to successfully develop a ZTB roadmap since these institutions jointly: (1) provide global leadership for TB prevention, care and control (WHO); (2) is responsible for improving animal health and welfare (OIE); and (3) work toward improving food security, nutrition and agricultural productivity and reduce rural poverty (FAO).

### Initial efforts to develop a ZTB roadmap

In March 2014, the ZTB sub-section created a working group to raise awareness of the public health risk posed by ZTB. This working group included participants from key parties including the:

- College of Veterinary Medicine and Biomedical Sciences, Colorado State University, United States of America
- Food and Agriculture Organization (FAO) of the United Nations with its Animal Production and Health Division
- International Union Against Tuberculosis and Lung Disease (The Union).
- Pan-American Foot-and-Mouth Disease Center (PANAFTOSA) from the Pan American Health Organization/Regional Office for the Americas of the World Health Organization
- Roslin Institute, Royal (Dick) School of Veterinary Studies, University of Edinburgh
- STOP TB Partnership
- United States Centers for Disease Control and Prevention (CDC)
- United States Department of Agriculture, Animal and Plant Health Inspection Service,
- World Health Organization (WHO) with its
  - Global TB Programme, and
  - The Department of Global Capacities, Alert, and Response
- World Organization for Animal Health (OIE)

One of the accomplishments of this working group was the publication of a manuscript (10) calling for a call to action in the Lancet Infectious Disease Journal.

A constellation of events has occurred to bring ZTB to the awareness of scientists, policymakers, government officials and the general public.

### May 2014: the end TB strategy

In May 2014, the World Health Assembly, WHO's yearly gathering of the world's Ministers of Health, approved the new post-2015 Global TB Strategy. The strategy aims to end, rather than merely control, the global TB epidemic, with targets to reduce TB deaths by 95% and to reduce new cases by 90% between 2015 and 2035, and to ensure that no family is burdened with catastrophic expenses due to TB. It set interim milestones for 2020, 2025, and 2030 (16). Thus, finding and treating every case of TB, whether caused by *M. tb* or *M. bovis*, will count toward the achievement of this ambitious goal. For this reason, as countries move toward detecting the 3 million TB cases estimated to be missed annually, and in light of the endorsed WHO “END TB” strategy, the Tripartite, The Union and the key organizations concerned with human and animal health, agriculture and TB joined forces to develop a Zoonotic TB Road Map outlining medium- and long-term milestones to globally address the prevention, surveillance, diagnostic, and treatment challenges faced by persons with ZTB.

### Sept 2015

In September 2015, the United Nations declared the end of the Millennium Development Goals and used them as the foundation for the Sustainable Development Goals (SDGs) (17) for 2015–2030, encompassing 17 broad and comprehensive topic areas, ranging from elimination of poverty and hunger, improved education and gender equality, to clean water and energy, action on climate and improvement of life under water and on land. The third SDG addresses global health, with TB highlighted as one of the priorities, thus presenting a key opportunity to improve the health of communities affected by ZTB.

### Nov 2015

The Stop TB Partnership, the global advocacy organization for TB, published the 4th edition of its Global Plan to End TB, 2016–2020 entitled “The paradigm shift” (18). This is a costed plan that includes actions needed to decrease TB, and is in full support of WHO's End TB Strategy. The Global Plan has set its 90-(90)-90 targets, which are to: identify 90% of all TB cases; concentrate on identifying 90% of those in key populations; and ensure 90% obtain appropriate treatment until cure. For the first time in this document, communities and people at risk of contracting ZTB were included as a key population.

### April 2016

A meeting co-organized by WHO and The Union, with contributions from leading international organizations for human and animal health, academic institutions, and non-governmental organizations took place at WHO in Geneva. There, the first steps toward formally conceptualizing a roadmap for ZTB began, in which ten priorities were identified to be presented to WHO's Global TB Programme's Strategic Technical Advisory Group (STAG) for TB in June 2016. In addition to the 10 institutions initially working on increasing global awareness of ZTB, the following institutions joined the efforts at this specific meeting:

- Swiss Tropical and Public Health Institute, Switzerland
- Animal and Plant Health Agency (APHA), United Kingdom
- University of Ibadan, Nigeria
- The Global Research Alliance for bovine Tuberculosis (GRAbTB)

The involvement and participation of GRAbTB was an important addition and played a strategic role in partnering with colleagues focusing in improve the understanding and control of bovine TB and developing novel and improved tools to control the disease at its bovine source (13). GRAbTB was established in 2014 and as part of its strategic goals, the alliance seeks not only to enhance collaboration within its members and institutions, but also with the broader human and animal TB research community.

### June 2016

The 10 priorities proposed for the ZTB roadmap were endorsed by the STAG, and a working group was created and tasked to produce and publish a ZTB Road Map during 2017.

#### ZTB patient testimonies

At the STAG meeting, a former ZTB survivor, from the Masaai community in Kenya shared with the scientific community the challenges she faced while suffering from this disease, which included: initial misdiagnosis, development of extrapulmonary (abdominal) TB, antimicrobial resistance to anti-TB drugs additional to the inherent resistance *M. bovis* has against pyrazinamide, and the need for longer (12 months) antimicrobial treatment, compared to the standard of 6 months.

During the 47th Union World Conference on Lung Health in Liverpool, England, a British veterinarian and former ZTB patient/survivor also shared his experience and challenges of initial misdiagnosis, extrapulmonary (pleural) TB, drug resistance to isoniazid in addition to pyrazinamide, and the longer treatment required while battling ZTB in the year 2013.

These patients' testimonies further emphasized that ZTB is not a disease from the past, and highlighted the challenges faced by certain communities at higher risk of contracting ZTB. Both patients emphasized the need of more awareness among the medical community to better diagnose and treat ZTB patients, and thus prevent the additional complications they had to endure.

### July 2017

The Heads of State comprising the G20 forum declared that “Shaping an interconnected world, calls for a One Health approach to tackling the spread of antimicrobial resistance and highlighted the need to foster research and development for TB” (19).

### October 2017

The Roadmap for ZTB was published and launched at the Union World Conference for Lung Health in Guadalajara, Mexico. The roadmap is the product of efforts of the tripartite partnership on Zoonotic Diseases comprising WHO, OIE, and FAO and The Union, and is available in English, French, and Spanish (13).

#### The role of media

over the past 3 years, all efforts, activities, and events related to ZTB were highlighted by a considerable number of local, regional, national, and global media sources including Central News Network (CNN), the British Broadcasting Corporation (BBC), and Le Monde newspaper in France, to name a few.

The Zoonotic TB Roadmap (13) outlines 10 priorities to address the existing challenges posed by ZTB, divided into three major core themes: (1) Improve the scientific evidence, (2) Reduce transmission at the animal-human interface, and (3) Strengthen intersectoral and collaborative approaches. Point 9 in the roadmap under core theme 3 specifically highlights the need of implementing integrated approaches to disease monitoring linking animal and public health (Box 1).

Box 1 **Zoonotic tb roadmap (13) point 9**

“Identify opportunities for community-tailored interventions that jointly address human and animal health Interventions that jointly address human and animal health can increase health and economic benefits for communities. Sharing of human resources, equipment and transport across sectors can reduce operational costs. This increased cost-effectiveness is especially relevant given the public funding constraints that often exist in settings where people are most at risk of zoonotic TB. For example, outreach childhood immunization campaigns or other existing livestock vaccination or testing programmes conducted in rural communities could be used to concurrently deliver educational and behavior change messages about food safety, to test livestock for bovine TB or, potentially in the future, to implement livestock vaccination campaigns against bovine TB. Interventions must be tailored to the cultural and socioeconomic characteristics of each setting. Community-driven participatory initiatives are key to achieving sustainability.”

### Moving forward

Although the publication of the ZTB Roadmap represents an unprecedented and historical accomplishment in the fight against global TB (20), there is still much work to be conducted in order to implement the actions needed to improve the prevention, diagnosis, control and treatment of ZTB. Table 1 is an excerpt from the ZTB Roadmap in which the short (year 2020) and medium term (year 2025) milestones to be accomplished are outlined under the three core themes of the roadmap. One of the key elements toward accomplishing these goals is that the unique cultural and socioeconomic factors that shape the relationship between people, livestock, and wildlife species in different ecosystems must be taken into account, while including the at- risk communities in future efforts to reduce the risk of zoonotic transmission of *M. bovis* across species. These efforts not only need to focus on preventing transmission from livestock (mostly cattle) to humans, but also, and in parallel, to reduce the prevalence of the disease in both domestic and wildlife species. The availability of improved diagnostic tools for ZTB in different species, as well as the implementation of disease monitoring, surveillance, and prevention strategies in livestock and wildlife species will be a crucial and much needed component to be able to implement comprehensive programs that will account for the complexities ZTB poses due to its zoonotic nature. Finally, including vaccination of wildlife (where feasible), not only has the potential for reducing the burden of disease, but also could play an important role in conservation efforts, especially among endangered and protected species.

**Table 1:** Timeline for action and milestones to be achieved in the short (2020) and medium term (2025) outlines in the ZTB Roadmap (13).

**Improve the scientific evidence base**	**Reduce transmission at the animal-human interface**	**Strengthen intersectoral and collaborative approaches**
**MILESTONES TO BE ACHIEVED BY YEAR 2020**		
Joint guidance developed for surveillance and management of zoonotic and bovine TB, at global and national levels	Capacity of national veterinary services strengthened for improving animal health, including detecting and controlling bovine TB in livestock and wildlife	Zoonotic and bovine TB properly addressed by government authorities and other stakeholders, in light of available evidence
Improved detection, recording and reporting of zoonotic and bovine TB within countries to allow more accurate estimations of disease burden	Efforts scaled-up to improve national food safety standards	Intersectoral and multidisciplinary collaborations established to build mechanisms and policies for One Health coordination and communication, within and between countries
Capacity of national healthcare and laboratory services strengthened for diagnosing and treating zoonotic TB	Community education campaigns implemented nationally to raise awareness of foodborne diseases and promote behavioral change	Global case for investment and business plan developed, providing rationale for investing in zoonotic and bovine TB and detailing the activities and resources needed
	Targeted surveys conducted to identify high-risk populations	Global advocacy strengthened to promote a research agenda that addresses knowledge gaps
**MILESTONES TO BE ACHIEVED BY YEAR 2025**		
New, rapid diagnostic tools available for diagnosing zoonotic TB and rolled-out to high risk groups	New diagnostics assays available for livestock	Mainstreaming of One Health approaches into efforts to improve human and animal health at global, national and community levels
Appropriate drug regimens defined for effective treatment of zoonotic TB	Effective bovine TB vaccines available for livestock and rolled-out in endemic settings	
Anti-TB vaccine available for people and rolled-out	Multi-species transmission pathways and sources of infection better characterized and used to inform the design of appropriate interventions	

## Conclusion

Implementing an integrated approach to develop the ZTB road map did not come without the inherent challenges of multidisciplinary and multi-institutional projects. That said, the accomplishment of this milestone in the fight against TB was the vision of a world free of TB, no matter what its source, the strong support of WHO and the Stop TB Partnership, a motivated core group that drove the process by providing clear goals and timelines, being inclusive by inviting all interested parties, and fostering a strong commitment to work together from colleagues and institutions both from the human and animal sectors that previously were working in isolation. This collaboration has opened new doors and opportunities as the world fights to end TB.

## Author contributions

All authors listed have made a substantial, direct and intellectual contribution to the work, and approved it for publication.

### Conflict of interest statement

The authors declare that the research was conducted in the absence of any commercial or financial relationships that could be construed as a potential conflict of interest.
